# Anterior Medial Meniscal Root Tears: A Novel Arthroscopic All Inside Repair

**Published:** 2014-09-01

**Authors:** L. Osti, A. Del Buono, N. Maffulli

**Affiliations:** 1 Unit of Arthroscopy and Sports Medicine, Hesperia Hospital, Modena, Italy.; 2 Department of Orthopaedics and Traumatology, Sant’Anna Hospital, Via Ravona, San Fermo della Battaglia (Como), Italy.; 3 Department of Musculoskeletal Disorders, Faculty of Medicine and Surgery, University of Salerno, Salerno, Italy;; 4 Centre for Sports and Exercise Medicine, Barts and The London School of Medicine and Dentistry, Mile End Hospital, 275 Bancroft Road, London E1 4DG, England.

## Abstract

**Background:**

Management of tears of the anterior and posterior roots of the meniscus is still controversial. We wish to propose a simple technique of suture anchor to repair tears of the anterior root of the medial meniscus.

**Methods:**

Twelve patients, active males, underwent arthroscopic repair of the anterior meniscal horn between 2009 and 2011. All were assessed postoperatively at an average follow-up of 1 year after the index operation.

**Results:**

At the last appointment, the average Lysholm scores was improved from a pre-operative average value of 48±17 to a postoperative value of 91±7 (P<0.001); five patients (45.3%) were scored as excellent (≥95), and 7 (54.6%) as good (85–94). At the last appointment, 8 of 9 active patients practiced sport at the same preoperative level, 1 (8.5%) had changed to lower level of activity. No technique related complications were evident.

## Background

Management of tears of the anterior and posterior roots of the meniscus is still controversial^1^. The posterior meniscal root is an anchor which maintains the circumferential hoop tension and prevents meniscal extrusions^2,3,4^. When injured, biomechanical and degenerative changes may occur, comparably to what occurs after total meniscectomy^5,6^. Therefore, surgical repair is advocated^7^. Even though arthroscopic all-inside techniques such as pull-out suture and suture anchors have been successfully used to address these lesions^4,8,9,10,11^, these procedures are technically demanding, especially if considering that even minimal shifts to the insertion site of the root may change the biomechanics, and induce to develop degenerative changes in the long term. On the other hand, the evidence in literature about the occurrence and management of meniscal tears within the anterior root, often undiagnosed, is still lacking. We wish to propose a simple technique of suture anchor to repair tears of the anterior root of the medial meniscus.

## Surgical Principles And Objectives

The congruity between menisci and cartilage is needed for proper load distribution^12,13^. When the anterior or posterior roots are disrupted, compartment pressures are abnormal, and menisci may be extruded^5,7^. We describe a relatively simple and safe procedure to repair anterior root meniscal tears, which does not interfere with concomitant procedures to the knee. For example, when this technique is used combining an ACL reconstruction, it does not produce any impairment on the tunnel drilling. This technique is minimally invasive, bone-sparing, and aims to restore the normal architecture and function of menisci. Surrounding hypertrophic synovial tissues are removed to improve the arthroscopic view, better visualize the lesion in its entirety, and to repair it in an anatomical fashion. An additional portal, proximal to the standard anteromedial portal, is usually used to assist and facilitate the passage of the suture within the tissue. In this way, it is possible to respect the insertion angle of the anchor and suture the lesion when the fat pad is hypertrophic. To provide stability and avoid excessive tension over the construct, the meniscus should be repaired when reduced, ensuring a good coverage of the tibial surface. Even though the method we use to assess meniscal features is anecdotal and based on surgical experience, we are able to restore the anatomical footprint without damages to the articular cartilages. The tension of the construct is tested intra-operatively, palpating the final repair with a probe, also in flexion and extension of the knee.

## Advantages

- Restore anatomy and biomechanics of the knee;
- Use of an additional portal to better handle the meniscus and visualize the lesion;
- Use of the arthroscopic setting needed for arthroscopic repair of shoulder instability

## Disadvantages

- Iatrogenic lesions;
- Need of advanced arthroscopic skills

## Indications

- Arthroscopic diagnosis of tear of the anterior root of the medial meniscus;
- Lack of coverage of the anterior tibial plate area by the anterior horn of the medial meniscus in the last few degrees to full extension of the knee (dynamic intra-operative evaluation). Even though the reliability of MRI is well known as the standard for assessment of soft tissues within and around the knee, the normal anatomy and coverage of the tibial plateau may be better examined under arthroscopy: the static evaluation is made with the knee flexed at 90°, the dynamic assessment is undertaken moving the knee from 90° of flexion to 0° of extension.
- Recurrent pain and discomfort to the knee mostly referred to the anteromedial area of the knee. It should be differentiated from an hypertopyc sinovial tissue of the anteromedial gutter, the only plausible condition of antero-medial pain and discomfort.

## Controindications

- Systemic disease;
- Neuromuscular disorders;
- Anatomic deformities and fractures to the knee;
- Narrowing of the medial rim of the joint (Rosenberg x-rays view);
- Previous surgery to the knee;
- Multiple ligament injury;
- Obesity. This condition can highly increase the risk of failure of the suture, replacement, and reinsertion of the meniscus.

## Patient information

- General risk factors related to arthroscopic surgery: infection, complex regional pain syndrome, deep vein thrombosis, pulmonary embolism, neurovascular iatrogenic injuries, failure;
- Possibility of iatrogenic damage to the infra-patellar branch of the safenus nerve. The risk is increased as a larger incision is needed to insert a portal working cannula;
- Prolonged rehabilitation protocol: full extension is allowed immediately, flexion is limited to 90° for 2 weeks, to 100° for 4 weeks and to 120° for 6 weeks;
- Clinical assessment at 3, 6, 9, and 12 months;
- A surgical failure may require a open procedure;
- Suture and anchor mobilization;
- Estimated stay in hospital 1 day. In Italy, based on the National Health Care System (NHCS) statement, patients undergoing ACL reconstruction require 1–2 days of hospitalization; patients undergoing procedures isolated to menisci and cartilage need 1 day of hospitalization.
- Running and squatting exercise are started at 3 months;
- Return to work activities, depending on the type of work.

## Preoperative Work Up

- History
- Clinical assessment;
- Antibiotic prophylaxis (single shot);
- MRI assessment of the knee;
- Anterior-posterior, lateral and Rosenberg x-rays views.

## Surgical Instruments and Implants

- 6–8 mm arthroscopic cannula;
- Anchor sutures;
- 19 gauge spinal needle;
- Arthroscopic grasper;
- Suture passer

## Anesthesia and Positioning

- Regional anaesthesia (block of sciatic, femoral, and obturator nerves). Spinal anesthesia may be done in almost all instances; general anestesia is undertaken successively when the pain is not properly controlled.
- Supine position;
- Non-sterile thigh tourniquet;
- Lateral post positioned at mid-thigh level.

## Surgical Technique

Figure 1 to 10

The procedure starts with the patient supine, with a non-sterile thigh tourniquet applied to the upper third of the thigh, and a lateral post positioned at the middle thigh level.

## Postoperative Management

- Wound dressing until the second postoperative day;
- Partial weight-bearing walking, range of motion exercise, quadriceps and hamstring strengthening exercises are allowed from the first postoperative day;
- Full extension is allowed immediately, flexion is limited to 90° for 2 weeks, 100° up to 4 weeks, and 120° up to 6 weeks; hyperextension or forced assisted extension should be avoided, proprioception loading exercises should be included.
- Running and squatting exercises are started after 3 months from the index procedure.

## Results

Twelve patients (right knee: 7 patients, left knee: 5 patients; average age 27, range 17–42) active males underwent arthroscopic repair of the anterior meniscal horn between 2009 and 2011. Associated injuries were identified, and treated accordingly in 7 patients: radiofrequency ablation of grade I and II cartilage lesions was made in 4 patients, microfractures of grade III and IV cartilage lesions in 3, limited lateral retinaculum release in 2, and ACL reconstruction in 2. All were assessed postoperatively at an average follow-up of 1 year after the index operation.

At the last appointment, the average Lysholm scores was improved from a pre-operative average value of 48±17 to a postoperative value of 91±7 (P<0.001). According to the Lysholm knee scoring system, five patients (45.3%) were scored as excellent (≥95), and 7 (54.6%) as good (85–94), without any significant difference between patients with and without cartilage lesions. Preoperatively, all patients were rated as C or D at IKDC assessment; at the latest follow-up, all of them were A and B. Symptoms were persistent in 1 patient (8.5%) who presented meniscal root pathology and a grade 2 lesion of the cartilage. After 4 months, he underwent a second look arthroscopy: residual fibrotic tissues were removed, with complete symptom resolution of symptoms at the latest follow-up. At the last appointment, 8 of 9 active patients practiced sport at the same preoperative level, 1 (8.5%) had changed to lower level of activity. No technique related complications were evident, and no patients experienced infection, paraesthesia or thrombosis after operation or limited range of motion over time.

## Figures and Tables

**Figure 1:**
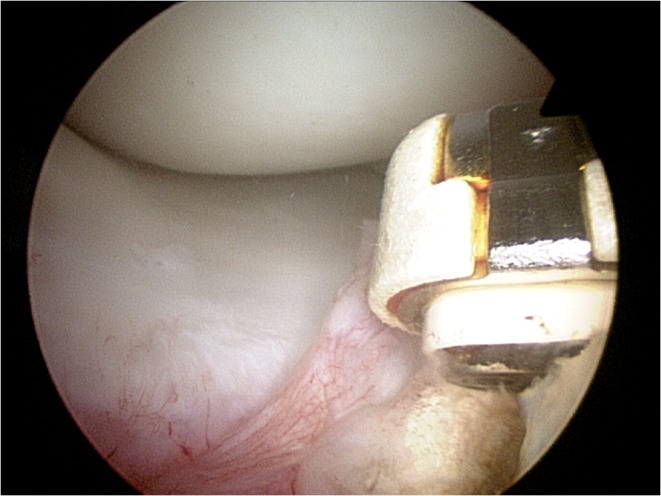
Diagnostic arthroscopy is performed through standard antero-lateral and antero-medial portals. Meniscal coverage over the anterior tibial plateau is assessed with the knee in flexion and extension. Once the surrounding hypertrophic synovial tissues have been removed, the lesion of the medial anterior horn is identified with the arthroscope in the standard anterolateral portal portal.

**Figure 2:**
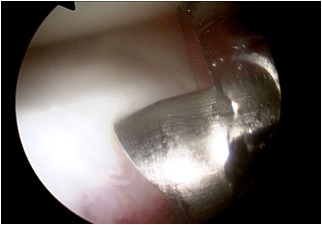
A 8 mm arthroscopic cannula is introduced through the antero-medial portal to improve suture passage. An accessory portal, proximal and medial to the main portal, can also be produced to assist and facilitate the passage of the sutures trough the meniscus.

**Figure 3–4:**
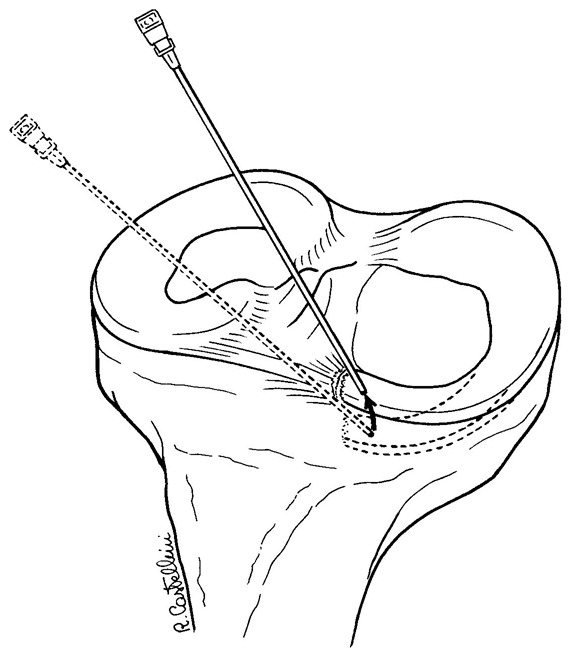

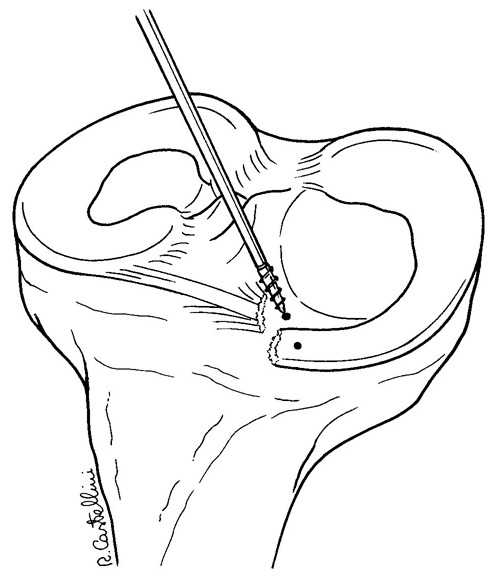
Remnant tissue are removed at the root attachment site. The anterior horn of the meniscus is carefully reduced with a 19 gauge spinal needle, and then with an arthroscopic grasper, assessing quality and tension of the meniscus at the new insertion site.

**Figure 5:**
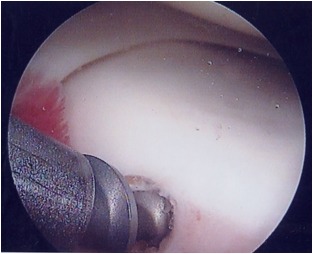
After careful debridement of the underside aspect of the meniscal root and surrounding capsular tissues, one anchor angled 45° in relation to the articular tibial surface is inserted, using as a guide a spinal needle pointing to the exact insertion site. Care is taken to avoid damage to the articular joint. The meniscal root is grasped and held with a suture passer, few millimeters from the edge of the root tear.

**Figure 6:**
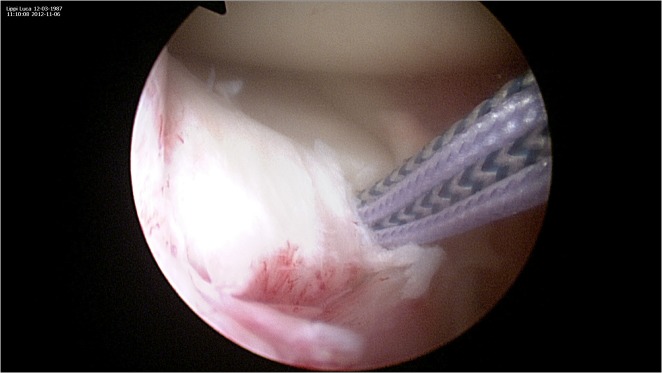
The anchor can be metallic or bio-absorbable, with single or double loaded sutures. The suture can be retrieved through an accessory supero-medial portal.

**Figure 7:**
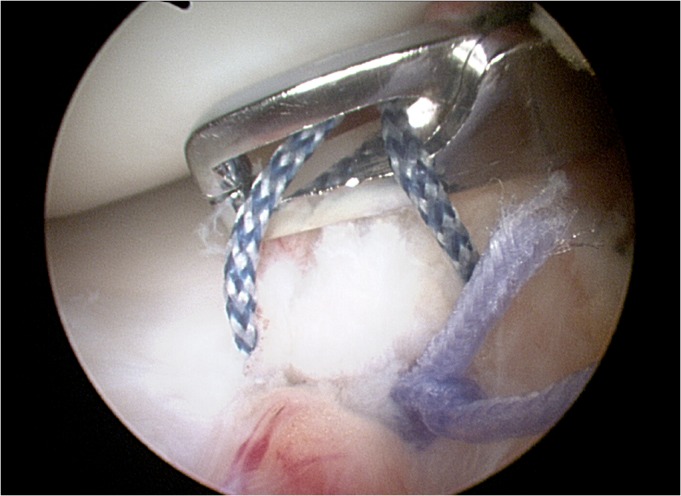
The passer is then advanced within the meniscal root, passing the suture within the meniscal tissue, and pulled out through the proximal superomedial portal. The other end of the non-absorbable suture is also shuttled within the root, and a mattress-suture repair is performed.

**Figure 8:**
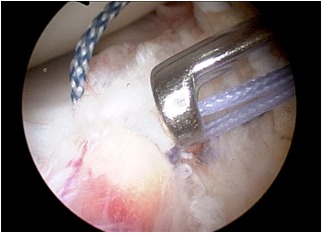
Sutures are tensioned keeping the meniscal root into or just over the repair socket, taking care to maintain the knot antero-inferior to the joint line and the area of tissue contact in the final phase of extension

**Figure 9:**
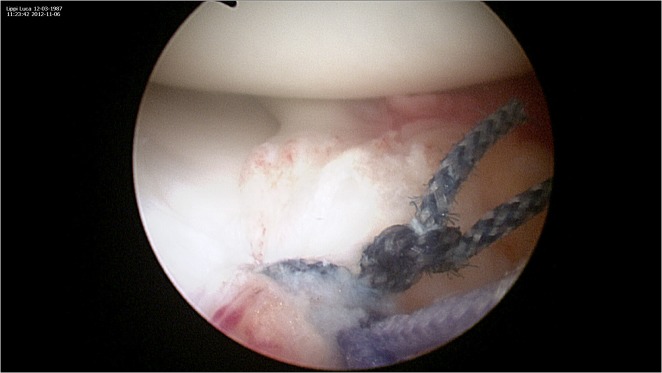
Once the reduction of the anterior horn has been tested, the sutures are tied over the tibial surface using a sliding low profile knot.

**Figure 10:**
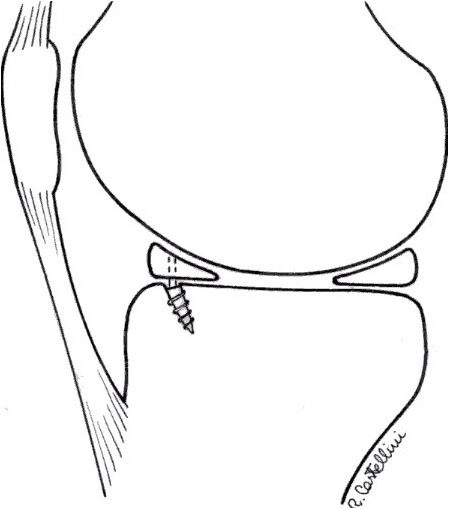
The stability of the repair and the juxtaposition of the construct to its anatomical insertion site are checked and tested with the knee in full extension.
